# Explaining biomass growth of tropical canopy trees: the importance of sapwood

**DOI:** 10.1007/s00442-015-3220-y

**Published:** 2015-01-30

**Authors:** Masha T. van der Sande, Pieter A. Zuidema, Frank Sterck

**Affiliations:** 1 Forest Ecology and Forest Management Group, Wageningen University, PO Box 47, 6700 AA Wageningen, The Netherlands; 2 Alterra—Wageningen University and Research Centre, PO Box 47, 6700 AA Wageningen, Netherlands; 3 Instituto Boliviano de Investigación Forestal, Casilla, 6204 Santa Cruz, Bolivia

**Keywords:** Bolivia, Carbon economy, Functional traits, Sapwood area, Sapwood turnover

## Abstract

Tropical forests are important in worldwide carbon (C) storage and sequestration. C sequestration of these forests may especially be determined by the growth of canopy trees. However, the factors driving variation in growth among such large individuals remain largely unclear. We evaluate how crown traits [total leaf area, specific leaf area and leaf nitrogen (N) concentration] and stem traits [sapwood area (SA) and sapwood N concentration] measured for individual trees affect absolute biomass growth for 43 tropical canopy trees belonging to four species, in a moist forest in Bolivia. Biomass growth varied strongly among trees, between 17.3 and 367.3 kg year^−1^, with an average of 105.4 kg year^−1^. We found that variation in biomass growth was chiefly explained by a positive effect of SA, and not by tree size or other traits examined. SA itself was positively associated with sapwood growth, sapwood lifespan and basal area. We speculate that SA positively affects the growth of individual trees mainly by increasing water storage, thus securing water supply to the crown. These positive roles of sapwood on growth apparently offset the increased respiration costs incurred by more sapwood. This is one of the first individual-based studies to show that variation in sapwood traits—and not crown traits—explains variation in growth among tropical canopy trees. Accurate predictions of C dynamics in tropical forests require similar studies on biomass growth of individual trees as well as studies evaluating the dual effect of sapwood (water provision vs. respiratory costs) on tropical tree growth.

**Electronic supplementary material:**

The online version of this article (doi:10.1007/s00442-015-3220-y) contains supplementary material, which is available to authorized users.

## Introduction

Tropical forests cover about 10 % of the earth’s surface, but store 25 % of global terrestrial carbon (C) and account for 34 % of terrestrial gross primary productivity (Bonan 2008; Lewis et al. 2009; Malhi 2012). They therefore feature prominently in climate change mitigation policies, such as reduced emissions from deforestation and forest degradation (REDD+) (Houghton 2005; Bonan 2008). In these forests, the 2 % largest stems account for at least 27 % of the aboveground biomass (Clark and Clark 1996; Lindenmayer et al. 2012; Slik et al. 2013). Since absolute biomass growth often increases with tree size (Clark and Clark 1999; Stephenson et al. 2014), the growth of large canopy individuals may largely determine the total aboveground C sequestration per ground area. Although several studies have evaluated the effect of environmental conditions and functional traits on diameter growth rates (Hérault et al. 2011), or on growth for small trees and saplings (Poorter 1999; Sterck et al. 2003), the understanding of what drives the biomass growth of individual canopy trees is still very poor.

The growth of a tree is affected by its ontogenetic stage, biotic and abiotic environment, and functional traits. Most studies, however, do not consider the direct relation between biomass growth and factors driving this at the individual tree level, but rather focus on average species performance and average species traits (e.g., Poorter and Bongers 2006; Wright et al. 2010). Yet, as Clark et al. (2011) pointed out, “individuals are the objects responding to environmental gradients, not species.” Species-specific performance of canopy trees may be partly driven by species-specific life history traits that allow them to endure in the understory and eventually reach the canopy. Still, variation among individuals may be substantial (Paine et al. 2011; Thomas et al. 2013) and important for their ecological performance (Violle et al. 2007) and contributions to population growth (Zuidema et al. 2009). Hence, individual tree-level analyses may yield important insights into the drivers of tree growth (Binkley et al. 2010; Sterck and Schieving 2011; Clark et al. 2011).

Functional traits are expected to link environmental conditions to growth, and may therefore assist in developing a mechanistic understanding of factors that drive tree growth (McGill et al. 2006; Ordoñez et al. 2009). Many studies have highlighted the importance of leaf traits, such as the positive effect of specific leaf area (SLA) and leaf nitrogen (N_leaf_) on growth of saplings and small trees (Wright et al. 2005; Sterck et al. 2006; Poorter and Bongers 2006). However, these relationships are generally weak for large trees, possibly because size-related traits such as total leaf area (TLA) may determine absolute tree growth more strongly than leaf traits (Poorter et al. 2008; Wright et al. 2010). In addition, stem traits also potentially affect whole-tree growth (Chave et al. 2009). An important stem-related trait is the sapwood area (SA) of a tree, which may indirectly increase photosynthesis rates by sustaining water transport to the leaves (Meinzer et al. 2008). However, extra SA may also incur additional maintenance respiration costs [see Meir and Grace (2002) for a positive effect of stem diameter on respiration], counterbalancing the positive water-related effect on growth (Wullschleger et al. 1998). So far, the contributions of size- and tissue-related stem and crown traits to individual growth of tropical canopy trees are poorly understood.

In this study we evaluated the relative effect of various size- and tissue-related stem and crown traits on biomass growth of 43 tropical canopy trees belonging to four species. Specifically, we ask the question: to what extent can variation in biomass growth across individual canopy trees be explained by crown and stem traits? We expected a positive relation between biomass growth and crown traits: TLA increases total light capture, a higher SLA increases the leaf area per unit biomass investment, and a higher N_leaf_ may increase the photosynthetic capacity (Poorter and Bongers 2006; Reich 2012). Furthermore, we expected that the sapwood N concentration (N_sapw_) would negatively affect growth, because high levels of N in wood would increase respiration. We did not have an a priori hypothesis about the relation between SA and tree growth, since the possible positive effects by augmenting water transport and storage might be offset by the negative effects of greater respiration loads.

## Materials and methods

### Research site

This study was conducted in the moist, semi-evergreen forest of La Chonta, Bolivia (15°47′S, 62°55′W). This is a 100,000-ha forestry concession that was established in 1974, with an average density of 367 trees per ha (>10 cm diameter at breast height) and a species richness of about 59 ha^−1^ (Peña-Claros et al. 2008). The average canopy height is 25 m, and most canopy trees have an estimated age of at least 150 years (Poorter and Bongers 2006; Rozendaal and Zuidema 2011). The average annual temperature is 24.3 °C and annual precipitation is 1,520 mm, with a dry season from April until September.

### Tree selection

From early April until early June 2012, forty-three emergent canopy trees were measured from four species representing different families and ecological growth strategies (Table 1): 15 individuals of *Hura crepitans*, 11 of *Schizolobium parahyba*, nine of *Cariniana ianeirensis* and eight of *Sweetia fruticosa*. Hereafter, these species will be referred to by their genus name. Moreover, these species were selected because they were known to produce distinguishable annual growth rings (Lopez et al. 2012). We selected trees with undamaged and fully exposed crowns and no or little liana cover. This ensured that growth differences among study trees were not strongly determined by differences in light availability. All measurements were conducted within hours after the selected trees were felled.

**Table 1:** The four species used in this study with family, guild, maximum tree height, average crown exposure index as juvenile (*CE*_juv_; value between 1 and 5 indicating increasing access to direct light), and average wood density (*WD*; g cm^−3^) at breast height

Species	Family	Guild	Maximum height	CE_juv_	WD
*Schizolobium parahyba*	Fabaceae/Caesalpiniaceae	LLP	35	2.39	0.45
*Sweetia fruticosa*	Fabaceae/Papillionaceae	LLP	30	1.91	0.82
*Hura crepitans*	Euphorbiaceae	PST	44	1.62	0.37
*Cariniana ianeirensis*	Lecythidaceae	PST	45	1.74	0.36

Long-lived pioneers (*LLP*) are long-lived species that need high irradiance to establish, and partially shade tolerant trees (*PST*) are species that can establish under low irradiance. WD data are obtained from this study; guild, maximum height and CE_juv_ are obtained from Poorter et al. (2006)

### Biomass growth

Directly following felling, we cut two stem discs using a chainsaw. One disc was obtained at about 1 m from the stem base and one just below the first major branch (between 6 and 17 m from the stem base). Bark thickness of the discs was measured in four directions, and the distances from the soil to the first disc and from the soil to the second disc were measured using a measuring tape. The discs were brought to the laboratory where they were polished to identify ring boundaries. On these discs, the radial length of the heartwood, sapwood and pith diameter were measured at the longest radius, the shortest radius, and one intermediate radius, using a caliper and a ruler. In all species except *Cariniana*, the distinction between sapwood and heartwood was clear, with abrupt switches in contrasting colors. For *Cariniana* sapwood area (SA) could therefore not be measured.

Per disc, ring width of the last 5 years was measured at the longest and shortest radius (using the pith as the center) and at one intermediate radius between the longest and shortest radii, since the discs were never fully a circle with the pith exactly in the center. We measured ring width using the TSAP-Win 0.53 software. The measurements of the three radii and of the 5 years were averaged to obtain one value for annual ring width per tree. We based our growth estimates on an average of the last 5 years, to minimize the effect of climatic variability on the growth estimates. Based on this average annual ring width and the diameter of the disc, the annual basal area (BA) growth was calculated.

At the same two heights per tree, 3- to 4-cm wide sections were cut in radial direction, from the bark to the pith. The bark was removed and the section was cut in a radial direction in samples of 6 cm, starting from the youngest sapwood until the pith was included. For each sample, fresh volume was determined using the water displacement method, and dry mass was measured after oven drying at 70 °C until the dry mass was stabilized. Wood density (WD; g cm^−3^) was calculated per wood sample by dividing the dry mass by the fresh volume.

In Supplementary Material Appendix 1 we show that, for our trees, taper only occurred between breast height and the first branch (i.e., along the main stem). We therefore calculated biomass growth separately for the stem (until the first branch) and crown. First, WD of the youngest sapwood was multiplied by the annual BA growth of the same disc to get a measure for the annual biomass growth per unit tree height (kg m^−1^ year^−1^), which could later be multiplied by height (separately for the stem and crown, as explained below) to obtain total biomass growth. To determine stem biomass growth, we assumed that the averaged biomass growth of the two disc samples was a good representation of the average biomass growth along the whole length of the main stem, until the first branch. Averaged biomass growth of the disc samples was subsequently multiplied by stem height to obtain an estimate of absolute stem biomass growth (kg year^−1^). To determine growth of woody biomass in the crown, we assumed that the biomass growth of the disc below the first branch was a good representative of the biomass growth of the whole crown. This biomass growth was multiplied by the length of the crown (maximum tree height minus stem height), measured with a laser rangefinder (Nikon Forestry 550), to obtain crown biomass growth (kg year^−1^). Note that we did not include leaf mass, as this strongly correlates with the total leaf area (TLA), which we used as one of the explanatory variables. Stem and crown biomass growth were subsequently summed to obtain an estimate of absolute aboveground biomass growth rate (kg year^−1^; Table 2). We chose this approach to calculate biomass rather than the more generally used allometric biomass equations, because it accounts for possible species-specific tapering within trunk and crown. As such, it likely provides a more direct and more reliable estimate of biomass than one based on generic biomass equations that are commonly used. We do acknowledge, though, that this is still an estimate of biomass (growth), which could be further refined, for example by using more detailed information on trunk tapering or WD variation along the stem.

**Table 2:** List of variables with abbreviation, units, mean, minimum, maximum, SD and coefficient of variation (*CV*)

Abbreviation	Variable description	Unit	Mean	Minimum	Maximum	SD	CV
AGR	Absolute biomass growth rate	kg year^−1^	105.43	17.32	367.3	80.68	0.77
Height	Tree height to top of crown	m	26.22	21.6	32.4	3.03	0.12
TLA	Total leaf area of the crown	m^2^	1,339.73	293.96	3,641	759.23	0.57
SA	Sapwood area	m^2^	0.172	0.029	0.577	0.119	0.69
SLA	Specific leaf area	cm^2^ g^−1^	105.65	72.6	149.7	17.76	0.17
N_leaf_	Leaf nitrogen concentration	%	2.56	1.82	3.42	0.43	0.17
N_sapw_	Sapwood nitrogen concentration	%	0.25	0.11	0.47	0.09	0.36
BA	Stem basal area	m^2^	0.331	0.096	0.838	0.183	0.55
Sapwood lifespan	Age of the sapwood	year	29.78	5.75	88.64	21.77	0.73
Sapwood growth	BA growth of 1 year	cm^2^ year^−1^	101.37	12.05	332	76.54	0.76

### Total leaf area

Per tree, we selected four to five undamaged branches that had a stem diameter of 4–8 cm and were growing in different parts of the crown. For each branch, all the apices with leaf-bearing shoots were counted. Then, for five randomly selected apices, the number of leaves was counted and one leaf was randomly selected and harvested. We thus obtained 20–25 leaves per tree. We pooled these leaves to measure the average leaf area (without petioles), using a desktop scanner. At the lower end of each branch, a disc was cut from which BA excluding bark was determined.

Per branch, the TLA was calculated by multiplying the number of shoots, the average number of leaves per shoot, and the average leaf area (obtained at the tree level). The ratio of cross-sectional BA to leaf area was determined per branch and averaged over four to five branches to obtain one value per tree.

To estimate TLA (m^2^), we assumed that the stem BA just below the first branch is proportional to its supporting leaf area. We tested this assumption by comparing the ratio of leaf area to BA at four sampling heights in the tree (see Supplementary Material Appendix 1 for details): breast height (1) just below the first branch (2) and at two heights in the crown below the lowest leaves (3 and 4). BA just below the first branch did not differ from the two upper sampling heights, supporting our assumption of a constant ratio between BA and leaf area just below the first branch and in the crown (see Supplementary Material Appendix 1). Therefore, we calculated TLA by dividing BA just below the first branch by the ratio BA:leaf area calculated from the branches of the same individual.

### Other traits

Per tree, the leaf area of the 20–25 pooled leaves was divided by their pooled dry mass (oven-dried at 70 °C until their mass was stabilized) to determine specific leaf area (SLA; cm^2^ g^−1^), and leaf samples per tree were analyzed for N concentration (N_leaf_; %, Table 2). The youngest wood samples at the two heights along the stem were pooled per tree and analyzed for N concentration (N_sapw_; %). SA (m^2^) per disc was determined by subtracting the heartwood and pith area from the total stem BA. SA per tree was calculated as the average SA of the discs taken at the two heights. Sapwood growth was defined as the annual BA growth (see “Biomass growth”), and sapwood lifespan was based on the number of annual rings in the sapwood. We estimated the number of annual rings in the sapwood by dividing the width of the sapwood by the average ring width of the last 15 years.

### Statistical analyses

For *Cariniana*, we could not distinguish sapwood from heartwood on the disc samples, so SA could not be measured. We carried out two sets of statistical analyses: one without *Cariniana* and one that included *Cariniana*, in which SA for *Cariniana* was predicted based on a regression analysis of SA versus all traits and BA of the other three species. These two approaches yielded similar results in terms of strength, direction and significance of coefficients of variables included in tests explaining variation in absolute biomass growth (see Supplementary Material Appendix 2). As including estimated SA values for *Cariniana* did not affect results, we present results of tests including *Cariniana* in the main text.

Our main aim was to evaluate how traits of individual trees could explain variation in their growth, and not the mean effect of species per se. To account for variation in growth that is explained by species’ differences, we included species as a fixed factor in the analyses. Growth, BA and SA were log transformed and TLA was square root transformed to meet the assumptions of equal variances and a normal distribution of the residuals. Possible interactions between species and one of the traits were first checked and included in further analyses if significant. Possible outlying observations were analyzed by applying Cook’s distance to the linear models.

The model including all traits, species, and interactions was reduced using all subsets regression analysis, which evaluates all possible combinations of predictor variables (Burnham and Anderson 2002). We used this technique because various combinations of variables in multiple regression models can give comparable good fits (Burnham and Anderson 2002; Johnson and Omland 2004). We therefore selected and averaged the models that differed less than 2 Akaike information criteria (AIC) units from the model that was selected as “best”. In this way, we obtained rather conservative but more robust model coefficients compared to what we would have obtained by selecting only the best model.

All analyses were performed using R 2.15.2. We used the following functions: lm for linear models, dredge for all subsets regression analysis, and model.avg for averaging regression models (the latter two from the MuMIn package; Barton 2012).

## Results

**Fig. 1:**
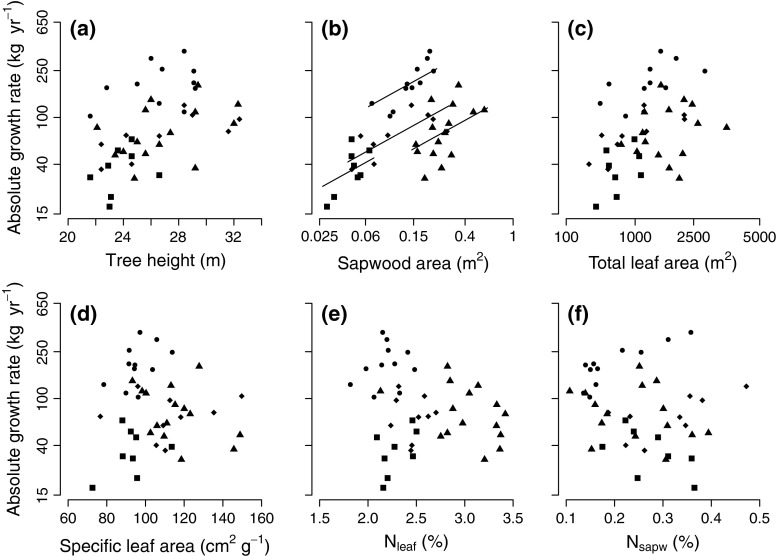
The relation of absolute biomass growth with **a** tree height, **b** sapwood area (SA), **c** total leaf area (TLA), **d** specific leaf area, **e** leaf nitrogen concentration (N_leaf_), and **f** sapwood N concentration (N_sapw_). *Regression lines* are based on the multiple regression analysis (by keeping the other predictor variables at their mean), but are only shown when the predictor variable contributed significantly in explaining absolute biomass growth (Table 3). Symbols represent four species: *Sweetia* (*squares*), *Hura* (*triangles*), *Schizolobium* (*circles*), and *Cariniana* (*diamonds*). Note that the axes for absolute biomass growth and SA have a log scale, and the axis for TLA a square root scale

The aboveground absolute growth rate (hereafter referred to as “growth”) ranged widely, between 17.32 and 367.25 kg year^−1^, with an average of 105.43 kg year^−1^ (Table 2). Many variables differed strongly among individuals and species, which can be seen from their high coefficient of variation. The averaged model, which included all variables, shows that only SA had a significant positive effect on growth (standardized coefficient = 0.73) and species differed in their intercept (Table 3; Fig. 1). The relative importance of SA and species on growth was 1 (i.e., the maximum) for both, and there were no significant interaction effects (species × traits). After SA, TLA had the strongest standardized coefficient, followed by SLA, N_sapw_, N_leaf_ and height (0.17, −0.16, 0.13, −0.12, and 0.11, respectively). The presented averaged model reflects the average of the five best-fitting models, because they differed less than two AIC units from the single best model.

**Table 3:** Results from the two linear models with absolute growth rate (*Growth*) and SA as response variables. The standardized coefficient (*β*), adjusted SE (*SEadj*), *t-*value, *P*-value, and relative variable importance [by summing the Akaike weights for all models where the specific variable was included (Barton 2012)] are given for each predictor variable. The effects on growth were evaluated by all subset regression analyses and subsequent averaging of the five models with Akaike information criteria values that differed by less than 2 units, therefore relative variable importance values could be obtained. The statistics of SA, however, were based on the full model (hence, no model averaging was applied and thus no relative variable importance values were calculated), based on variables scaled by subtracting the mean and dividing by the SD. Note that *Cariniana* was excluded from the analysis for SA

Response variable	Predictor variable	*β*	SEadj	*t*-value	*P*-value	Relative importance
Growth	log (SA)	0.73	0.15	4.68	<0.001	1
	Intercept *Sweetia*	0	0			1
	Intercept *Hura*	−0.28	0.19	1.41	0.158	
	Intercept *Schizolobium*	0.56	0.14	3.93	<0.001	
	Intercept *Cariniana*	0.07	0.12	0.56	0.574	
	SLA	−0.16	0.10	1.60	0.111	0.56
	N_sapw_	0.13	0.08	1.47	0.142	0.33
	Sqrt (TLA)	0.17	0.11	1.46	0.146	0.14
	Height_max_	0.11	0.10	1.03	0.304	0.08
	N_leaf_	−0.12	0.14	0.84	0.401	0.07
SA	BA	0.22	0.07	3.32	0.002	
	Sapwood growth	0.45	0.08	5.99	<0.001	
	Sapwood lifespan	0.18	0.07	2.50	0.019	
	Intercept *Sweetia*	−0.77	0.12	−6.55	<0.001	
	Intercept *Hura*	1.42	0.16	8.67	<0.001	
	Intercept *Schizolobium*	0.50	0.20	2.52	0.018	

Relative importance was given for the variable species, therefore no importance value is shown for the intercepts of the individual species

We evaluated the robustness of our results by adding a number of analyses, of which results are included in the Appendices (2, 4, 5 and 6). First of all, we evaluated the results with different proxies for tree size, i.e., tree height or BA. Results of statistical analyses showed that SA and species were the most significant predictor variables, irrespective of the tree size proxy used (cf. Supplementary Material Appendix 2a with Table 3). We continued using tree height as size proxy, since this correlated more weakly than BA with most of the other predictor variables of growth (*r* < 0.6 for tree height, see Supplementary Material Appendix 3, and *r* < 0.86 for BA), suggesting that the effects of tree height on growth were independent of impacts by other crown or stem trait. Second, for sake of comparison, we present the analysis of the effect of traits on BA growth (cf. Supplementary Material Appendix 4 with analysis for absolute biomass growth in Table 3), which showed that traits similarly affect both growth measures. We further focused on biomass growth and not BA growth or stem diameter growth, as biomass growth is most relevant for C sequestration. Third, in addition to our all subset regression analysis and model averaging, we added an analysis for biomass growth using the standard stepwise exclusion of variables, and showed that SA and species were the most significant predictor variables in both analyses (cf. Supplementary Material Appendix 5 with Table 3). Last, we performed an analysis using a reduced model, in order to evaluate results for a pre-selected limited set of variables. The model in which only tree height, TLA and SA were included as explanatory variables again confirmed that SA and species were the only variables explaining variation in biomass growth (cf. Supplementary Material Appendix 6 with Table 3). The results of the analysis presented in Table 3 are thus in line with a number of alternative analyses presented in Supplementary Material Appendices (2, 4, 5, and 6).

**Fig. 2:**
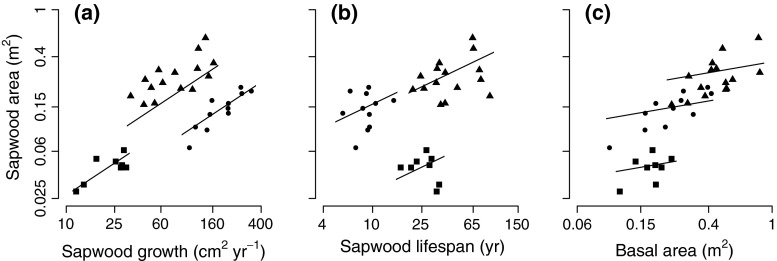
The relation of sapwood area (SA) with **a** sapwood growth, **b** sapwood lifespan, and **c** stem basal area (BA). *Regression lines* are based on the multiple regression analysis (by keeping the other predictor variables at their mean), but are only shown when the predictor variable contributed significantly in explaining absolute biomass growth (Table 3). Symbols represent four species: *Sweetia* (*squares*), *Hura* (*triangles*) and *Schizolobium* (*circles*). *Cariniana* was excluded because no SA could be distinguished. Note that the axes for SA and stem BA have a log scale

Because SA was the most important explanatory variable for growth, we elaborated further on factors that may explain variation in SA. We evaluated how SA depends on SA growth, sapwood lifespan and stem BA. In this analysis, SA growth, i.e., newly formed SA per year, ranged between 12.05 and 332.00 cm^2^ year^−1^ with an average of 101.37 cm^2^ year^−1^, sapwood lifespan ranged between 5.7 and 88.6 year with an average of 29.78 year, and BA ranged between 0.10 and 0.83 m^2^ with an average of 0.33 m^2^ (Table 2). We included species as fixed factor (species did not interact with other predictor variables), and scaled all numeric variables by subtracting the mean and dividing by the SD, to obtain standardized coefficients. The results showed that sapwood growth, sapwood lifespan, and stem BA all positively affected SA, with standardized coefficients of 0.45, 0.18, and 0.22, respectively (Table 3; Fig. 2).

## Discussion

Our aim was to explain variation in absolute biomass growth (referred to as “growth”) among individual tropical canopy trees by stem and crown traits. From all traits, sapwood area (SA) turned out to be the only variable that significantly increased with growth (Table 3; Fig. 1). Growth was not affected by tree height or basal area (BA), indicating that size does not drive differences in growth among canopy trees. Further evaluation of factors explaining variation in SA across trees showed a positive effect of sapwood growth, sapwood lifespan and tree BA on SA (Table 3; Fig. 2).

### An individual-based approach

We used an individual-based approach to evaluate the factors driving variation in growth among tropical forest canopy trees. By combining individual traits and species in one statistical model, we were able to separate the effect that individual traits have on individual growth from the variation caused by evolutionary differences among species (Clark et al. 2011). Our focus is on individuals because they are the units that grow and respond to their environment (Clark et al. 2011), rather than species. While other studies show that differences in growth and other traits among individuals of the same species even exceed the differences in average growth or traits among species (Bolnick et al. 2003; Clark 2010; Messier et al. 2010), this was not the case in our study. Possible explanations are that we used four species with different ecological growth strategies, and selected fully exposed canopy trees with reduced environmental variation among individuals. Nevertheless, we observed fully consistent trait impacts on growth among individuals, suggesting that similar functional relationships drive the growth variation among individuals for different species.

### Sapwood is the major driver of growth, not crown traits

Contrary to expectations, we found that none of the traits, except for SA, explained variation in growth of individual canopy trees. Many studies have found an important positive role of leaf traits such as total leaf area (TLA), specific leaf area (SLA) and leaf nitrogen concentration (N_leaf_) for species performance (Sterck et al. 2006, 2014), especially for saplings and small trees (Poorter 1999; Poorter and Bongers 2006). These traits indeed vary strongly among species and partially explain species-level growth responses of smaller trees, where a high TLA, SLA and N_leaf_ may strongly increase the light interception and photosynthesis per unit plant mass and therefore drive growth. The importance of such crown traits may be different for canopy trees that have full access to light and better developed crowns, with optimally distributed leaves that compensate for possible effects of leaf traits such as SLA and N_leaf_ on the light capture and C gain (McMurtrie et al. 2008; Sterck and Schieving 2011). Similar to our results, Staudhammer et al. (2013) found no effect of TLA on basal area (BA) growth of adult trees (although TLA did increase reproductive output). Thus, crown traits cannot explain the variation in stem growth among emergent tropical canopy trees.

**Fig. 3:**
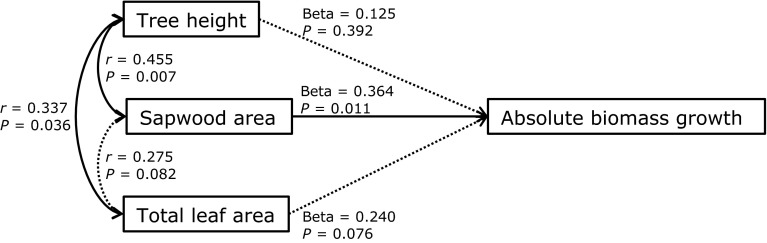
Structural equation model for the effects of tree height, sapwood area (SA) and total leaf area (TLA) on absolute biomass growth. For each variable, the species mean was subtracted from the individual measurements in order to exclude differences in intercept among species, as were found in previous analysis (Table 3). The *one-headed arrows* show regressions between variables, whereas the *two-headed arrows* between the predictor variables show correlations between variables. *Black arrows* show significant effects and *dotted arrows* show non-significant effects. For each relation, the coefficient (*β* or *r*) and significance (*P*) are given, based on an *n* of 43. Note that the model is saturated (i.e., all possible arrows between boxes are drawn), therefore we cannot test the fit of the overall model. We nevertheless present this model in order to evaluate the relative strengths of size variables on growth while correcting for interrelatedness among predictor variables. The model was evaluated using the sem function of the lavaan package in R (Rosseel 2012)

SA was clearly the most important variable explaining aboveground biomass growth of individual trees in our study. A high amount of living wood may increase respiration costs (Ryan et al. 1994), especially when air temperature is high, and have a negative effect on growth. Interestingly, a positive effect of SA was superior to the increase in respiration costs (Table 3; Fig. 1), probably because tall trees can be water limited and SA improves the water supply to the crown. This relation could not be explained by larger trees that have both a high biomass growth and large SA, since growth rate was not related to tree height (Table 3; Fig. 2) nor was it related to BA (see Supplementary Material Appendix 2a). We added a structural equation model (Fig. 3) to summarize the relative effects of SA, TLA and tree height on growth when taking correlations among predictors into account. Even though the effect of TLA on growth was marginally significant as compared to the linear model (Table 3), the analysis confirmed that SA is superior to any other effect on growth.

### Growth and sapwood: chicken and egg?

A question that arises from the positive relation between SA and growth, is whether sapwood has a positive functional effect on growth, or is merely a passive consequence of growth (Galván et al. 2012)? In other words, does a large SA increase growth, or does fast growth increase SA? To better understand these relations, we evaluated some factors that may explain variation in SA. A tree can have a lot of sapwood because of fast sapwood growth, long sapwood lifespan, and/or because the tree has a large BA and consequently a large SA. We found that all these three factors positively affect SA (Table 3; Fig. 2). The positive effect of BA on SA indicates that larger trees have more SA, but BA did not affect growth (see Supplementary Material Appendix 5). The positive effects of sapwood growth and sapwood lifespan on SA (Table 3; Fig. 2) suggest that trees can achieve a larger SA by increasing sapwood growth and/or sapwood lifespan. However, the negative correlation between sapwood growth and sapwood lifespan (see Supplementary Material Appendix 4) suggests that trees with fast sapwood growth, which increases SA, also have a short sapwood lifespan, which decreases SA. Hence, the SA should not necessarily increase as a result of tree growth. Moreover, since the average sapwood lifespan is 30 years, average annual sapwood growth should at least be an order of magnitude smaller than the total SA of the tree. Hence, it is unlikely that this small part of the SA that is directly related to annual growth causes the strong positive relation between SA and growth. These results imply that SA is not only a passive consequence of growth, but that the positive effect of SA on growth may be attributed to a functional role of sapwood underlying growth.

### Why does SA increase growth?

**Fig. 4:**
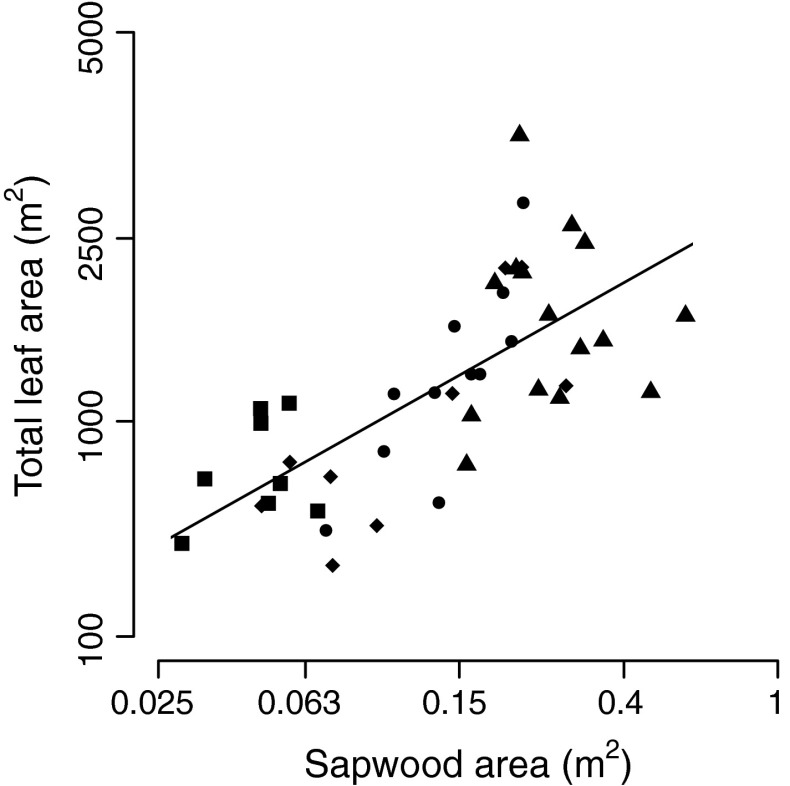
The relation of sapwood area (SA) with total leaf area (TLA), based on a regression analysis. Symbols represent four species: *Sweetia* (*squares*), *Hura* (*triangles*), *Schizolobium* (*circles*), and *Cariniana* (*diamonds*). Note that the axis for SA has a log scale and the axis for TLA a square root scale

The functional role of sapwood is to supply water with nutrients to the crown, and this is likely how SA increases biomass growth in our study trees. Sapwood assures water supply in two ways: by water transport from the roots to the leaves (Goldstein et al. 1998; Meinzer et al. 2001), and by water storage to buffer the use of soil water and allow more persistent water supply to the crown during the course of the day (e.g., during hot afternoons) or the dry season (Wullschleger et al. 1998). Our canopy trees were all emergent and thus most likely not primarily limited by light, but their high stature (on average 26.2 m) may have caused hydraulic limitation for the supply of water to the crown. We found a positive effect of SA on TLA (Fig. 4), without differences in slope and intercept between species. This suggests that a large SA indeed supports a large TLA, and that, independent of species, a certain SA is associated with a certain TLA. A positive relation between SA and TLA was also found for two mountain ash species in south-east Australia (Vertessy et al. 1995), and a strong relation between SA and water flow rate was found for five tropical canopy trees in Panama (Goldstein et al. 1998). These studies and our results thus suggest that the water supply to the crown may limit the TLA and growth of these tropical forest trees.

The sapwood age (i.e., sapwood lifespan) of our trees ranged between 5.7 and 88.6 years with an average of 29.8 years (Table 2). We did not find other studies with data on sapwood lifespan for tropical trees, but Spicer and Holbrook (2007) found ages between 7.6 and 50 years for three temperate tree species, and Sterck et al. (2008) found ages between 25 and 50 years for *Pinus sylvestris* (a coniferous species) in an alpine valley. Compared to these studies, trees in our study varied strongly in sapwood lifespan, with some having remarkably old sapwood. Since water transport efficiency decreases with sapwood age (Spicer and Gartner 2001), it is unlikely that all 30 years of the sapwood have an equally important contribution to water transport. Instead, the oldest sapwood rings may be used to store water and nutrients in living cells and extracellular spaces (Goldstein et al. 1998), rather than to transport water. Goldstein et al. (1998) found that the majority of the stored water in large trees was used in the morning to supplement water that had been lost through transpiration during the previous day, before the soil water could reach these depleted sites. The stored water may act as a buffer to complement water supply to the upper leaves, which reduces the risk on drought-induced cavitation of the vessels, and simultaneously increases photosynthesis by allowing more water to be withdrawn for transpiration (Scholz et al. 2007).

The whole-tree hydraulic conductance can be evaluated by using the ratio between TLA and SA. This ratio determines the water supply per unit leaf area and, hence, may affect actual rates of photosynthesis and growth (Whitehead et al. 1984; McDowell et al. 2002). For our trees, however, the ratio between SA and TLA did not relate to growth (linear model with species as fixed factor; *t* = −1.33, *P* = 0.891). Probably, the SA available per leaf is not a good indicator of water reaching the leaves for large trees, because of the reduced transport activity of the old sapwood. McDowell et al. (2002) showed that the ratio between leaf area and SA decreases with tree height, indicating that for large trees the hydraulic conductance becomes relatively less important than their capacity to store water (Phillips et al. 2003). Given the old age of the sapwood in our trees (5.7–88.6 year), the lack of effect of hydraulic conductance (the ratio between TLA and SA) on growth, and the expected hydraulic limitations during periods of low water availability, we speculate that an increased SA positively affects growth by improving water storage, rather than water transport.

We show that SA may be one of the most important traits affecting the growth of tropical canopy trees. Few studies have focused on the role of sapwood for biomass growth (but see Galván et al. 2012), and no studies have done so for tropical trees. Our results suggest that the positive functional effects of SA on growth largely offset possible negative impacts of increasing respiration costs. We speculate that this is attributable to an increasing capacity for water storage that sustains water supply to the leaves, even in times of high evaporative demand and/or drought.

#### **Author contribution statement**

MS, PZ and FS designed the study and fieldwork. MS performed the fieldwork. MS, PZ and FS analysed the data and wrote the manuscript.

## Electronic supplementary material

Below is the link to the electronic supplementary material.

Supplementary material 1 (DOCX 105 kb)
