# Supplementary material for: Explaining biomass growth of tropical canopy trees: the importance of sapwood
Source: Oecologia. 2015 Jan 30;177(4):1145–55. doi: 10.1007/s00442-015-3220-y (PMC4363484; doi:10.1007/s00442-015-3220-y)
Supplement: Supplementary file 1 — Supplementary material 1 (DOCX 105 kb) [file 442_2015_3220_MOESM1_ESM.docx]

**Appendices**

| **Appendix 1:** Results for the comparisons of basal area (in m^2^) among the four sampling heights in the tree: breast height (1), just below the first major branch (2), and at two heights in the crown below the first leaves (3 and 4; see figure on the right). For each height, the mean, minimum, maximum, and standard deviation (Stdev) of the basal area are given. A two-way ANOVA showed no interaction between species and height, and we therefore used a one-way ANOVA with TukeyHSD multiple comparisons to compare basal area at the four heights (letters indicate different groups at P < 0.001). N was 43 for all comparisons. | |
| --- | --- |
| \| Height \| Mean \| Min \| Max \| Stdev \| TukeyHSD* \| \| --- \| --- \| --- \| --- \| --- \| --- \| \| 1 \| 0.40 \| 0.13 \| 1.31 \| 0.24 \| a \| \| 2 \| 0.23 \| 0.06 \| 0.68 \| 0.13 \| b \| \| 3 \| 0.24 \| 0.04 \| 0.98 \| 0.18 \| b \| \| 4 \| 0.21 \| 0.03 \| 0.95 \| 0.17 \| b \| | 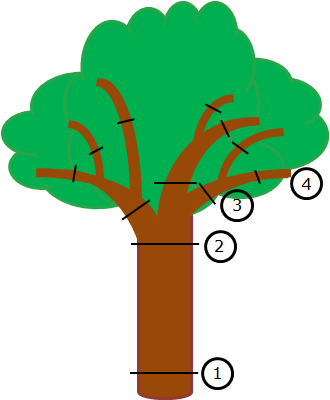 |

| **Appendix 2:** Evaluation of the effect on the analyses when (*a*) including predicted sapwood area for *Cariniana* (as used in the manuscript)*,* and (*b*) when excluding *Cariniana* from the analyses. All subset regression analyses were used with absolute growth rate as response variable and all traits, stem basal area and species as predictor variables, and model averaging was applied over all models that differed less than 2 AIC from the ‘best’ model (which is considered a not significantly different fit). Empty rows indicate that the variable was excluded by the analysis. These results also show the effect of including stem basal area instead of tree height (Appendix 2a vs. Table 3). The standardized coefficient (Beta), adjusted standard error (SEadj), z-value and P-value are given for each predictor variable. N = 43 for the model with predicted sapwood area for *Cariniana*, and N = 34 for the model without *Cariniana*. |
| --- |
| \|  \| *a)* Including Cariniana \| \| \|  \| *b)* Excluding Cariniana \| \| \|  \| \| --- \| --- \| --- \| --- \| --- \| --- \| --- \| --- \| --- \| \| Predictor variable \| Beta \| SEadj \| z-value \| P-value \| Beta \| SEadj \| z-value \| P-value \| \| log(SA) \| 0.74 \| 0.15 \| 4.77 \| <0.001 \| 0.91 \| 0.19 \| 4.71 \| <0.001 \| \| Intercept *Sweetia* \| 0 \| 0 \|  \|  \| 0 \| 0 \|  \|  \| \| Intercept *Hura* \| -0.28 \| 0.20 \| 1.44 \| 0.149 \| -0.54 \| 0.25 \| 2.19 \| 0.029 \| \| Intercept *Schizolobium* \| 0.56 \| 0.14 \| 3.94 \| <0.001 \| 0.40 \| 0.17 \| 2.39 \| 0.017 \| \| Intercept *Cariniana* \| 0.07 \| 0.13 \| 0.55 \| 0.585 \|  \|  \|  \|  \| \| SLA \| -0.16 \| 0.10 \| 1.60 \| 0.110 \| -0.13 \| 0.11 \| 1.22 \| 0.224 \| \| Nsapw \| 0.13 \| 0.09 \| 1.47 \| 0.142 \| 0.08 \| 0.09 \| 0.91 \| 0.365 \| \| sqrt(TLA) \| 0.17 \| 0.12 \| 1.46 \| 0.146 \|  \|  \|  \|  \| \| Nleaf \| -0.12 \| 0.14 \| 0.84 \| 0.401 \|  \|  \|  \|  \| |

| **Appendix 3:** Pearson correlations among *a)* the predictor variables used to explain variation among trees in absolute biomass growth, and *b)* the predictor variables used to explain variation among trees in sapwood area. |
| --- |
| \| *a)* \|  \|  \|  \|  \| *b)* \|  \|  \| \| --- \| --- \| --- \| --- \| --- \| --- \| --- \| --- \| \| log(SA) \| sqrt(TLA) \| SLA \| N_leaf_ \| N_sapw_ \| log(BA) \| log(sapwood growth) \|  \| \| 0.564 \| 0.461 \| 0.340 \| 0.325 \| -0.090 \|  \|  \| Height \| \|  \| 0.699 \| 0.474 \| 0.511 \| -0.204 \|  \|  \| log(SA) \| \|  \|  \| 0.500 \| 0.394 \| 0.090 \|  \|  \| sqrt(TLA) \| \|  \|  \|  \| 0.599 \| 0.095 \|  \|  \| SLA \| \|  \|  \|  \|  \| 0.007 \|  \|  \| N_leaf_ \| \|  \|  \|  \|  \|  \| 0.314 \|  \| log(sapwood growth) \| \|  \|  \|  \|  \|  \| 0.478 \| -0.547 \| log(sapwood lifespan) \| |

| **Appendix 4:** Results from the linear model for basal area growth, including the same predictor variables as used for absolute biomass growth (see Table 3). Only the predictor variables are presented that were included in the best models (based on all subset regression analysis), and statistics are based on averaging of these ‘best’ models (i.e., that differed less than 2 AIC units). The standardized coefficient (Beta), standard error (SE), *t*-value, *P*-value, and relative variable importance are given for each predictor variable. The statistics for the species are based on their intercept. |
| --- |
| \| **Predictor variable** \| **Beta** \| **SEadj** \| ***t*-value** \| ***P*-value** \| **Relative importance** \| \| --- \| --- \| --- \| --- \| --- \| --- \| \| log(SA) \| 0.56 \| 0.12 \| 4.73 \| <0.001 \| 1 \| \| Intercept *Sweetia* \| 0 \| 0 \|  \|  \| 1* \| \| Intercept *Hura* \| 0.15 \| 0.17 \| 0.91 \| 0.362 \|  \| \| Intercept *Schizolobium* \| 0.79 \| 0.11 \| 6.91 \| <0.001 \|  \| \| Intercept *Cariniana* \| 0.35 \| 0.10 \| 3.48 \| 0.001 \|  \| \| SLA \| -0.10 \| 0.08 \| 1.29 \| 0.199 \| 0.30 \| \| N_leaf_ \| -0.13 \| 0.12 \| 1.10 \| 0.272 \| 0.23 \| |

* Relative importance was given for the variable ‘species’. Therefore no importance value is shown for the intercepts of the individual species.

| **Appendix 5:** Results of the linear regression for absolute biomass growth, using stepwise exclusion of variables (based on AIC), in order to compare these with the results based on all subsets regression analysis and model averaging (Table 3). All traits, tree height and species were initially included as predictor variables. All continuous variables were scaled prior to analysis, by subtracting the mean and dividing by the standard deviation, to obtain standardized coefficients (Beta). Furthermore, standard error (SE), *t*-value and *P*-value are given for each predictor variable. The significance of the intercepts of the different species are relative to the intercept of *Sweetia*. |
| --- |
| \| **Predictor variable** \| **Beta** \| **SE** \| ***t*-value** \| ***P*-value** \| \| --- \| --- \| --- \| --- \| --- \| \| log(SA) \| 0.80 \| 0.14 \| 5.70 \| <0.001 \| \| SLA \| -0.15 \| 0.09 \| -1.63 \| 0.113 \| \| N_sapw_ \| 0.13 \| 0.08 \| 1.58 \| 0.124 \| \| Intercept *Sweetia* \| -0.15 \| 0.26 \| -0.60 \| 0.555 \| \| Intercept *Hura* \| -0.74 \| 0.38 \| -1.57 \| 0.126 \| \| Intercept *Schizolobium* \| 1.13 \| 0.30 \| 4.21 \| <0.001 \| \| Intercept *Cariniana* \| 0.00 \| 0.29 \| 0.52 \| 0.607 \| |

| **Appendix 6**: Results of the reduced linear model for aboveground biomass growth, including only sapwood area (SA), total leaf area (TLA), tree height and species as predictor variables (without exclusion of variables). All continuous variables were scaled prior to analysis, by subtracting the mean and dividing by the standard deviation, to obtain standardized coefficients (Beta). Furthermore, standard error (SE), *t*-value and *P*-value are given for each predictor variable. The significance of the intercepts of the different species are relative to the intercept of *Sweetia*. |
| --- |
| \|  \| **Beta** \| **SE** \| ***t*-value** \| ***P*-value** \| \| --- \| --- \| --- \| --- \| --- \| \| log(SA) \| 0.58 \| 0.19 \| 3.10 \| 0.004 \| \| sqrt(TLA) \| 0.08 \| 0.11 \| 0.70 \| 0.486 \| \| Height \| 0.08 \| 0.10 \| 0.76 \| 0.455 \| \| Intercept *Sweetia* \| -0.19 \| 0.28 \| -0.69 \| 0.493 \| \| Intercept *Hura* \| -0.71 \| 0.41 \| -1.26 \| 0.215 \| \| Intercept *Schizolobium* \| 1.13 \| 0.32 \| 4.17 \| <0.001 \| \| Intercept *Cariniana* \| -0.02 \| 0.30 \| 0.57 \| 0.573 \| |
